# Supplementary material for: Recognition of Rare Microfossils Using Transfer Learning and Deep Residual Networks
Source: Biology (Basel). 2022 Dec 21;12(1):16. doi: 10.3390/biology12010016 (PMC9854841; doi:10.3390/biology12010016)
Supplement: Supplementary file 1 [file biology-12-00016-s001.zip › biology-2031655-supplementary/6 Supplementary tables.pdf]

Supplementary tables

Table S1. Precision, recall and F1-score of nine microfossils using four training methods.

Training methods	Evaluation index	*Cono-theca*	*Archae-ooides*	*Quadra-pyrgites*	*Proto-hertzina*	*Maik-hanella*	*Carin-achites*	*Qin-scolex*	*Hyoli-thellus*	dross	Average Value
ResNet	Recall	1.00	0.83	1.00	0.80	0.88	1.00	0.86	0.81	0.48	0.851
	Precision	1.00	1.00	0.94	1.00	0.92	1.00	1.00	0.90	0.89	0.961
	**F1-score**	**1.000**	**0.907**	**0.969**	**0.889**	**0.900**	**1.000**	**0.925**	**0.853**	**0.624**	**0.896**
TF-ResNet	Recall	0.86	0.92	0.99	0.86	0.93	0.98	0.74	0.83	0.87	0.887
	Precision	1.00	0.95	0.97	1.00	0.94	0.99	1.00	0.88	0.78	0.946
	**F1-score**	**0.925**	**0.935**	**0.980**	**0.925**	**0.935**	**0.985**	**0.851**	**0.854**	**0.823**	**0.912**
**TS-ResNet**	Recall	1.00	0.95	1.00	0.93	0.99	1.00	0.95	0.99	0.98	0.977
	Precision	0.99	1.00	0.99	1.00	1.00	1.00	1.00	0.93	0.95	0.984
	**F1-score**	**0.995**	**0.974**	**0.995**	**0.964**	**0.995**	**1.000**	**0.974**	**0.959**	**0.965**	**0.980**
TA-ResNet	Recall	1.00	0.98	1.00	0.82	1.00	1.00	0.95	0.96	0.96	0.963
	Precision	1.00	1.00	0.98	1.00	0.97	1.00	1.00	1.00	0.99	0.993
	**F1-score**	**1.000**	**0.990**	**0.990**	**0.901**	**0.985**	**1.000**	**0.974**	**0.980**	**0.975**	**0.977**

Table S2. Precision, recall, and F1-score of nine microfossils in TS-ResNet.

Retrained layers in TS-ResNet	Evaluation index	*Cono-theca*	*Archae-ooides*	*Quadra-pyrgites*	*Proto-hertzina*	*Maik-hanella*	*Carin-achites*	*Qin-scolex*	*Hyoli-thellus*	dross	Average Value
conv5_x	Recall	0.99	0.92	1.00	0.80	1.00	1.00	1.00	0.97	0.95	0.959
	Precision	1.00	1.00	1.00	1.00	1.00	1.00	1.00	0.99	0.97	0.996
	**F1-score**	**0.995**	**0.958**	**1.000**	**0.889**	**1.000**	**1.000**	**1.000**	**0.980**	**0.960**	**0.976**
conv4_x-conv5_x	Recall	1.00	1.00	1.00	0.80	0.98	1.00	1.00	1.00	0.97	0.972
	Precision	1.00	1.00	1.00	1.00	0.88	1.00	1.00	0.99	1.00	0.986
	**F1-score**	**1.000**	**1.000**	**1.000**	**0.889**	**0.927**	**1.000**	**1.000**	**0.995**	**0.985**	**0.977**
**conv3_x-conv5_x**	Recall	1.00	0.95	1.00	0.93	0.99	1.00	0.95	0.99	0.98	0.977
	Precision	0.99	1.00	0.99	1.00	1.00	1.00	1.00	0.93	0.95	0.984
	**F1-score**	**0.995**	**0.974**	**0.995**	**0.964**	**0.995**	**1.000**	**0.974**	**0.959**	**0.965**	**0.980**
conv2_x-conv5_x	Recall	1.00	1.00	1.00	0.80	0.99	1.00	0.99	0.96	0.95	0.966
	Precision	0.98	1.00	1.00	1.00	0.98	1.00	1.00	0.97	1.00	0.992
	**F1-score**	**0.990**	**1.000**	**1.000**	**0.889**	**0.985**	**1.000**	**0.995**	**0.965**	**0.974**	**0.978**

Table S3. Influence of different numbers of training images on the performance of TS-ResNet.

Train data/Class	Evaluation index	*Cono-theca*	*Archae-ooides*	*Quadra-pyrgites*	*Proto-hertzina*	*Maik-hanella*	*Carin-achites*	*Qin-scolex*	*Hyoli-thellus*	dross	Average Value
200	Recall	1.00	0.95	1.00	0.93	0.99	1.00	0.95	0.99	0.98	0.977
	Precision	0.99	1.00	0.99	1.00	1.00	1.00	1.00	0.93	0.95	0.984
	**F1-score**	**0.995**	**0.974**	**0.995**	**0.964**	**0.995**	**1.000**	**0.974**	**0.959**	**0.965**	**0.980**
100	Recall	0.99	0.98	1.00	0.83	1.00	1.00	0.98	0.97	0.97	0.969
	Precision	1.00	1.00	0.98	1.00	1.00	1.00	1.00	0.94	0.97	0.988

	**F1-score**	**0.995**	**0.990**	**0.990**	**0.907**	**1.000**	**1.000**	**0.990**	**0.955**	**0.970**	**0.977**
50	Recall	0.97	1.00	1.00	0.80	1.00	1.00	0.96	0.91	0.93	0.952
	Precision	1.00	1.00	0.97	1.00	0.99	0.99	1.00	1.00	0.98	0.992
	**F1-score**	**0.985**	**1.000**	**0.985**	**0.889**	**0.995**	**0.995**	**0.980**	**0.953**	**0.954**	**0.971**
10	Recall	0.95	0.99	1.00	0.80	1.00	1.00	0.58	0.58	0.97	0.888
	Precision	1.00	0.80	0.97	1.00	1.00	0.99	1.00	1.00	0.72	0.942
	**F1-score**	**0.974**	**0.884**	**0.985**	**0.889**	**1.000**	**0.995**	**0.734**	**0.824**	**0.826**	**0.901**
5	Recall	0.98	0.98	1.00	0.81	0.95	1.00	0.55	0.65	0.90	0.869
	Precision	0.99	0.85	0.95	1.00	1.00	0.95	1.00	0.84	0.68	0.918
	**F1-score**	**0.985**	**0.910**	**0.974**	**0.895**	**0.974**	**0.974**	**0.710**	**0.733**	**0.775**	**0.881**
3	Recall	0.96	1.00	0.98	0.82	0.84	0.97	0.51	0.61	0.69	0.820
	Precision	0.98	0.83	0.96	1.00	1.00	1.00	1.00	0.69	0.68	0.904
	**F1-score**	**0.970**	**0.907**	**0.970**	**0.901**	**0.913**	**0.985**	**0.675**	**0.648**	**0.685**	**0.850**
1	Recall	0.62	0.99	0.74	0.64	0.41	0.93	0.20	0.33	0.67	0.614
	Precision	0.95	0.64	0.95	1.00	1.00	0.64	0.74	0.72	0.72	0.817
	**F1-score**	**0.752**	**0.774**	**0.832**	**0.780**	**0.582**	**0.756**	**0.315**	**0.452**	**0.694**	**0.660**

Table S4. Performance comparison of machine learning, deep learning methods and TS-ResNet.

Evaluation Metrics	Train data/Class	HOG+SVM	TS-VGG	TS-InceptionV3	**TS-ResNet**
Average F1-score	200	0.927	0.969	0.949	**0.980**
	10	0.560	0.883	0.797	**0.901**
	3	—	0.805	0.588	**0.849**
	1	—	0.640	0.416	**0.660**

Table S5. F1-score of machine learning, deep learning methods and TS-ResNet under nine microfossils.

Methods	Train data/Class	*Cono-theca*	*Archae-ooides*	*Quadra-pyrgites*	*Proto-hertzina*	*Maik-hanella*	*Carin-achites*	*Qin-scolex*	*Hyoli-thellus*	dross	Average F1-score
HOG+SVM	200	0.945	0.957	0.853	0.985	0.902	0.964	0.890	0.866	0.983	0.927
	10	0.561	0.583	0.472	0.618	0.543	0.605	0.538	0.495	0.625	0.560
	—	—	—	—	—	—	—	—	—	—	—
	—	—	—	—	—	—	—	—	—	—	—
TS-VGG	200	1.000	0.953	0.990	0.942	0.985	1.000	0.985	0.939	0.931	0.969
	10	0.937	0.891	0.860	0.895	0.814	0.961	0.901	0.792	0.896	0.883
	3	0.971	0.955	0.836	0.870	0.904	0.904	0.601	0.529	0.673	0.805
	1	0.731	0.902	0.447	0.571	0.804	0.699	0.593	0.281	0.733	0.640
TS-InceptionV3	200	0.995	0.870	0.990	0.889	0.990	1.000	0.985	0.959	0.862	0.949
	10	0.974	0.956	0.683	0.936	0.809	0.954	0.639	0.497	0.720	0.797
	3	0.817	0.339	0.355	0.932	0.630	0.892	0.540	0.123	0.667	0.588
	1	0.271	0.337	0.577	0.397	0.699	0.704	0.230	0.193	0.333	0.416
TS-ResNet	200	0.995	1.000	0.974	0.974	0.995	0.964	0.995	0.965	0.959	0.980
	10	0.974	0.884	0.985	0.889	1.000	0.995	0.734	0.824	0.826	0.901
	3	0.970	0.907	0.970	0.974	0.830	0.985	0.675	0.648	0.685	0.849
	1	0.752	0.774	0.832	0.780	0.582	0.756	0.315	0.452	0.694	0.660

Table S6. Precision, recall and F1-score of nine microfossils under TS-VGG, TS-InceptionV3 and TS-ResNet.

Methods/ Model depth	Train data/Class	Evaluation index	*Conotheca*	*Archaeooides*	*Quadrapyrgites*	*Protohertzina*	*Maikhanella*	*Carinachites*	*Qinscolex*	*Hyolithellus*	dross	Average Value
TS-VGG	200	Recall	1.00	0.91	0.98	0.89	0.98	1.00	0.97	0.93	0.88	0.949
		Precision	1.00	1.00	1.00	1.00	0.99	1.00	1.00	0.95	0.99	0.992
		**F1-score**	**1.000**	**0.953**	**0.990**	**0.942**	**0.985**	**1.000**	**0.985**	**0.939**	**0.931**	**0.969**
	10	Recall	0.89	0.98	0.89	0.81	0.72	0.98	0.82	0.76	0.91	0.862
		Precision	0.99	0.82	0.83	1.00	0.94	0.94	1.00	0.83	0.88	0.914
		**F1-score**	**0.937**	**0.891**	**0.860**	**0.895**	**0.814**	**0.961**	**0.901**	**0.792**	**0.896**	**0.883**
	3	Recall	0.99	0.96	0.94	0.77	0.85	0.89	0.43	0.37	0.75	0.772
		Precision	0.95	0.95	0.75	1.00	0.97	0.92	1.00	0.93	0.61	0.897
		**F1-score**	**0.971**	**0.955**	**0.836**	**0.870**	**0.904**	**0.904**	**0.601**	**0.529**	**0.673**	**0.805**
	1	Recall	0.85	0.88	0.38	0.40	0.76	0.57	0.49	0.17	0.65	0.572
		Precision	0.64	0.93	0.54	1.00	0.85	0.91	0.75	0.81	0.84	0.808
		**F1-score**	**0.731**	**0.902**	**0.447**	**0.571**	**0.804**	**0.699**	**0.593**	**0.281**	**0.733**	**0.640**
TS-InceptionV3	200	Recall	0.99	0.77	1.00	0.80	1.00	1.00	0.97	0.99	0.92	0.938
		Precision	1.00	1.00	0.98	1.00	0.98	1.00	1.00	0.93	0.81	0.967
		**F1-score**	**0.995**	**0.870**	**0.990**	**0.889**	**0.990**	**1.000**	**0.985**	**0.959**	**0.862**	**0.949**
	10	Recall	0.95	0.99	0.68	0.95	0.72	0.94	0.47	0.42	0.59	0.746
		Precision	1.00	0.93	0.69	0.92	0.92	0.9	1.00	0.61	0.92	0.884
		**F1-score**	**0.974**	**0.956**	**0.683**	**0.936**	**0.809**	**0.954**	**0.639**	**0.497**	**0.720**	**0.797**
	3	Recall	0.69	0.21	0.30	0.96	0.52	0.99	0.37	0.08	0.55	0.519
		Precision	1.00	0.88	0.44	0.91	0.80	0.81	1.00	0.27	0.85	0.771
		**F1-score**	**0.817**	**0.339**	**0.355**	**0.932**	**0.630**	**0.892**	**0.540**	**0.123**	**0.667**	**0.588**
	1	Recall	0.16	0.36	0.77	0.25	0.64	0.89	0.13	0.11	0.32	0.403
		Precision	0.89	0.32	0.46	0.96	0.77	0.58	1.00	0.79	0.35	0.679
		**F1-score**	**0.271**	**0.337**	**0.577**	**0.397**	**0.699**	**0.704**	**0.230**	**0.193**	**0.333**	**0.416**
TS-ResNet	200	Recall	1.00	1.00	0.95	0.95	0.99	0.93	1.00	0.98	0.99	0.977
		Precision	0.99	1.00	1.00	1.00	1.00	1.00	0.99	0.95	0.93	0.984
		**F1-score**	**0.995**	**1.000**	**0.974**	**0.974**	**0.995**	**0.964**	**0.995**	**0.965**	**0.959**	**0.980**
	10	Recall	0.95	0.99	1.00	0.80	1.00	1.00	0.58	0.70	0.97	0.888
		Precision	1.00	0.80	0.97	1.00	1.00	0.99	1.00	1.00	0.72	0.942
		**F1-score**	**0.974**	**0.884**	**0.985**	**0.889**	**1.000**	**0.995**	**0.734**	**0.824**	**0.826**	**0.901**
	3	Recall	0.96	1.00	0.98	0.95	0.71	0.97	0.51	0.61	0.69	0.820
		Precision	0.98	0.83	0.96	1.00	1.00	1.00	1.00	0.69	0.68	0.904
		**F1-score**	**0.970**	**0.907**	**0.970**	**0.974**	**0.830**	**0.985**	**0.675**	**0.648**	**0.685**	**0.849**
	1	Recall	0.62	0.99	0.74	0.64	0.41	0.93	0.20	0.33	0.67	0.614
		Precision	0.95	0.64	0.95	1.00	1.00	0.64	0.74	0.72	0.72	0.817
		**F1-score**	**0.752**	**0.774**	**0.832**	**0.780**	**0.582**	**0.756**	**0.315**	**0.452**	**0.694**	**0.660**
